# Traditional Biomolecular Structure Determination by NMR Spectroscopy Allows for Major Errors

**DOI:** 10.1371/journal.pcbi.0020009

**Published:** 2006-02-03

**Authors:** Sander B Nabuurs, Chris A. E. M Spronk, Geerten W Vuister, Gert Vriend

**Affiliations:** 1 Center for Molecular and Biomolecular Informatics, Nijmegen Center for Molecular Life Sciences, Radboud University, Nijmegen, Netherlands; 2 Department of Biophysical Chemistry, Institute for Molecules and Materials, Radboud University, Nijmegen, Netherlands; University of California San Diego, United States of America

## Abstract

One of the major goals of structural genomics projects is to determine the three-dimensional structure of representative members of as many different fold families as possible. Comparative modeling is expected to fill the remaining gaps by providing structural models of homologs of the experimentally determined proteins. However, for such an approach to be successful it is essential that the quality of the experimentally determined structures is adequate. In an attempt to build a homology model for the protein dynein light chain 2A (DLC2A) we found two potential templates, both experimentally determined nuclear magnetic resonance (NMR) structures originating from structural genomics efforts. Despite their high sequence identity (96%), the folds of the two structures are markedly different. This urged us to perform in-depth analyses of both structure ensembles and the deposited experimental data, the results of which clearly identify one of the two models as largely incorrect. Next, we analyzed the quality of a large set of recent NMR-derived structure ensembles originating from both structural genomics projects and individual structure determination groups. Unfortunately, a visual inspection of structures exhibiting lower quality scores than DLC2A reveals that the seriously flawed DLC2A structure is not an isolated incident. Overall, our results illustrate that the quality of NMR structures cannot be reliably evaluated using only traditional experimental input data and overall quality indicators as a reference and clearly demonstrate the urgent need for a tight integration of more sophisticated structure validation tools in NMR structure determination projects. In contrast to common methodologies where structures are typically evaluated as a whole, such tools should preferentially operate on a per-residue basis.

## Synopsis

Three-dimensional biomolecular structures provide an invaluable source of biologically relevant information. To be able to learn the most of the wealth of information that these structures can provide us, it is of great importance that the quality and accuracy of the protein structure models deposited in the Protein Data Bank are as high as possible. In this work, the authors describe an analysis that illustrates that this is unfortunately not the case for many protein structures solved using nuclear magnetic resonance spectroscopy. They present an example in which two strikingly different models describing the same protein are analyzed using commonly available structure validation tools, and the results of this analysis show one of the two models to be incorrect. Subsequently, using a large set of recently determined structures, the authors demonstrate that unfortunately this example does not stand on its own. The analyses and examples clearly illustrate that relying solely on the experimental data to evaluate structural quality can provide a false sense of correctness and the combination of multiple sophisticated structure validation tools is required to detect the presence of errors in protein nuclear magnetic resonance structures.

## Introduction

Experimentally determined three-dimensional structures of biomolecules form the foundation of structural bioinformatics, and any structural analysis would be impossible without them. Two main techniques are available for biomolecular structure determination: x-ray crystallography and nuclear magnetic resonance (NMR) spectroscopy. It is important to realize that all resulting structure models are derived from their underlying experimental data. Unfortunately, any experiment and thus any structure model will have errors associated with it. Random errors depend on the precision of the experimental measurements and are propagated to the precision of the final models. Systematic errors and mistakes often result from errors in the interpretation of the experimental data and relate directly to the accuracy of the final structure models. For example, in NMR spectroscopy errors can be introduced by misassignment of the spectral signals; in x-ray crystallography errors are most likely made when the protein structure is positioned in the electron density [1,2].

Several studies have shown that not all experimentally determined biomolecular structure models are of equally high quality [3–6]. Many different types of errors can be identified in protein structures, ranging from too tightly restrained bond lengths and angles, to molecules exhibiting a completely incorrect fold. Where the former type of errors often does not have large consequences for the analysis of the structure and typically can be easily remedied by refinement in a proper force field [7,8], the latter renders a structure model completely useless for all practical purposes. Throughout the years several such errors have been uncovered in the Protein Data Bank (PDB) [9], which often resulted in the replacement of the incorrect models with improved ones.

A typical example of an incorrectly folded structure model is the first crystal structure of photoactive yellow protein. The structure was solved initially in 1989 [10] and deposited under the now obsolete PDB entry 1PHY. An updated model released 6 y later showed that in the original model the electron density had been misinterpreted [11] (PDB entry 2PHY). Similar chain tracing problems led to an incorrect model for a DD-peptidase [12] (the now obsolete PDB entry 1PTE), which was corrected 10 y later when the structure was solved again but now at higher resolution [13] (PDB entry 3PTE).

Also, for structures determined using NMR spectroscopy, cases are known where reevaluation of the experimental data, often prompted by publication of a corresponding structure, has resulted in the replacement of structures in the PDB. A well-known example is the original NMR structure of the oligomerization domain of p53 [14]. In this dimer of dimers, a difference in the orientation of the two dimers was observed between the NMR and crystal structure, the latter published shortly after the NMR structure [15] (PDB entry 1C26). Reexamination of the nuclear Overhauser enhancement (NOE) data led to the identification of three misinterpreted peaks in the original p53 NOE assignments and the inclusion of several new NOEs, resulting in a revision of the original PDB entry [16] (PDB entry 1OLH). A similar low number of misinterpreted NOE signals (17 in total) resulted in a largely incorrect fold for the anti–σ factor AsiA [17] (the now obsolete PDB entry 1KA3). In this case, it was not until a second solution structure of AsiA was published [18] (PDB entry 1JR5) that the experimental data of the original AsiA structure were reexamined and the assignment errors were discovered [19] (updated PDB entry 1TKV).

In this paper, we describe a detailed analysis of two recently released NMR structures of the protein dynein light chain 2A (DLC2A), one from human (PDB entry 1TGQ) and one from mouse (PDB entry 1Y4O). Both structures originate from large structural genomics initiatives: the structure of human DLC2A (hDLC2A) was determined by the Northeast Structural Genomics Consortium (NESGC, http://www.nesg.org), and the mouse variant (mDLC2A) was determined by the Center for Eukaryotic Structural Genomics (CESG, http://www.uwstructuralgenomics.org). Despite 96% sequence identity, large structural differences are observed between the two ensembles; an unexpected and extremely unlikely result. Using the deposited experimental data we show that only the 1Y4O structure ensemble is correct. Subsequently, we analyze both ensembles using various structure and data validation methods to show that the erroneous structure ensemble could have been identified prior to deposition. Finally, we validate a large set of protein NMR structures that were released from the PDB in the period 2003 to 2005 and show that the DLC2A example does not stand on its own, but that more errors of this magnitude can be found. We conclude with some suggestions on how, in the future, such large errors can be identified during the structure determination process using readily available validation software.

## Results/Discussion

Our interest in DLC2A originated from a request by one of our collaborators to build a homology model for this protein. A BLAST search in February 2005 [20] against the PDB revealed that construction of a homology model should be straightforward: two NMR structures of DLC2A (PDB entries 1Y4O and 1TGQ), both with more than 95% sequence identity to the target sequence, had been released in the months prior to our query. Surprisingly, a first visual inspection of both structures revealed striking differences, as shown in Figure 1.

**Figure 1:**
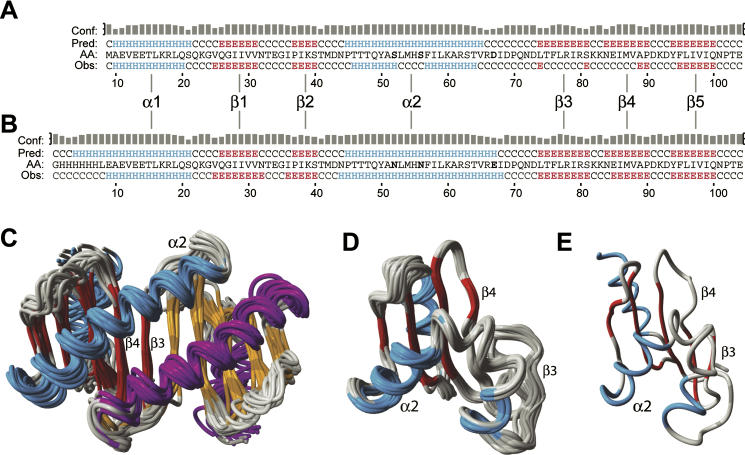
Sequence and Structure Ensembles of Two DLC2A Structures (A) The sequence of human DLC2A (hDLC2A) (AA). (B) The sequence of mouse DLC2A (mDLC2A) proceeded by an eight-residue His-tag (AA). The secondary structure as predicted using PSIPRED [33,50] (Pred) and the confidence of this prediction (Conf) are shown above the sequences. The secondary structure as observed in the ensembles (Obs) is indicated below the sequences. Except for the His-Tag, the mouse and human sequences differ at three positions (indicated in bold). (C) Ribbon diagram of the structure ensemble of mDLC2A (PDB entry 1Y4O). The residues of the His-tag have been omitted for clarity. (D) Ribbon diagram of the structure ensemble of hDLC2A (PDB entry 1TGQ). (E) The refined average structure of the ensemble calculated using the reconstructed 1TGQ dataset, as discussed in the text. Secondary structure is indicated using colors: helices are shown in blue and purple, strands are shown in red and orange. A numbering scheme for the secondary structure elements is indicated between the two sequences.

It is immediately obvious that DLC2A forms a dimer in the 1Y4O structure models (Figure 1C), whereas the 1TGQ ensemble contains DLC2A in monomeric form (Figure 1D). Additionally, the DLC2A models feature remarkably different folds. The central α-helix (α2 in Figure 1A and 1B), which extends from Asn44 to Ile68 in the 1Y4O ensemble, consists in the 1TGQ ensemble of two separate, almost antiparallel, α-helices (Thr46-Ser52 and Phe57-Thr64) connected by a turn-like region (Leu53-Ser56). Beta strands β3 (Leu71-Ser80) and β4 (Glu85-Pro90) pack tightly against each other in the 1Y4O structure models. In the 1TGQ structures, the β3 region forms a hairpin-like structure, and the β4 strand is much less tightly packed against the core of the protein.

During evolution, protein structure has always been more stable and has changed much slower than the associated sequence [21]. As a result, similar sequences fold into practically identical structures and remotely related sequences still adopt similar folds [22]. An accurate limit for this rule was recently derived by Rost [23], who found that two sequences that share over 30% sequence identity in 100 aligned residues are practically guaranteed to have the same fold. Given this knowledge, it is extremely unlikely for mouse and human DLC2A, which share 96% sequence identity, to fold into the different structures shown in Figure 1C and 1D.

Visual inspection of the two ensembles made us realize quickly that the large differences probably originate from the oligomeric state of the two structures. Using NMR spectroscopy (and in most structural genomics initiatives [24]), the presence of tertiary structure in a soluble protein is typically assessed using a proton-nitrogen correlation (^15^N-HSQC) spectrum [25]. The observed pattern of dispersed signals, ideally one for each amino acid, provides a “fingerprint” of the protein. However, the formation of a symmetric dimer, as shown in Figure 1A, does not result in a doubling of the number of observed NMR signals. Consequently, it is not straightforward to determine the oligomeric state of a protein from its ^15^N-HSQC NMR spectra alone, and typically assessments have to be made from estimates of the protein's relaxation rates [26]. Therefore, if the oligomeric state of a protein is not known or is incorrectly known, the NMR spectra of a dimeric protein could be easily interpreted as originating from a monomer. Below, we present evidence that such a misinterpretation is the root-cause of the observed differences between the human and mouse DLC2A structure ensembles.

Figure 1C shows that the two α2-helices in the dimer interface are oriented in an antiparallel fashion. As a result, intermolecular signals arising from, for example, contacts between the N-terminal and C-terminal sides of these respective helices are to be expected. When it is a priori known that the protein under investigation is a dimer, specific experiments can be performed to distinguish such intermolecular contacts from the intramolecular ones [27]. However, if the intermolecular contacts are wrongly interpreted as intramolecular, the residues involved would appear to be close to each other also in the monomeric structure, something that is indeed observed in the structure models shown in Figure 1D.

To further test this hypothesis, we used the experimental NMR restraints from the 1Y4O structure ensemble (as those for the 1TGQ ensemble were not available) and changed all 72 intermolecular NOEs into 36 intramolecular distance restraints. With this simulated subset of 36 erroneous intramolecular NOEs (hereafter referred to as the 1TGQ_sim_ dataset) and the experimentally observed intramolecular restraints, structure calculations were performed. An ensemble of 20 structures without any distance violations larger than 0.5 Å was readily obtained. The refined geometric average of this ensemble is shown in Figure 1E, and it exhibits a fold very similar to that observed for the 1TGQ ensemble. These results provide a strong indication that the NMR spectra of hDLC2A were indeed interpreted as those of a monomer, while the protein, like its mouse homolog, is actually a dimer in solution. Conclusive evidence that the human DLC2A protein does indeed form a dimer was obtained from the NESGC Web site, where the aggregation screening records associated with hDLC2A clearly show that this protein forms dimers in solution (http://spine.nesg.org/buffer_exchange.pl?id=HR2106). During the reviewing process of this paper, one of the referees pointed us to the publication of an independent structure determination of the human homolog in August 2005 (PDB entry 1Z09) [28], which was indeed also solved as a dimer. Subsequently, in November 2005, 1.5 y after its original deposition, the monomeric PDB entry 1TGQ was replaced by a correct dimeric structure (PDB entry 2B95).

### Data and Structure Analyses

Having established the origin of the errors present in the 1TGQ ensemble, we can now ask the most important question: Could these errors have been discovered during the structure determination and validation process? To investigate this issue, the deposited structure ensembles were evaluated using common structure validation tools. In addition, both structure ensembles were refined in explicit solvent [7,8] and subsequently also included in the structure validation process. The DLC2A models of the 1Y4O ensemble were refined against the deposited NOE distance restraints and dihedral angle restraints. As mentioned before, for the 1TGQ ensemble no experimental restraints had been deposited, and therefore the intramolecular restraints as obtained from the 1Y4O dataset were used. In addition, the restraints from the 1TGQ_sim_ dataset were also included in the refinement of the 1TGQ structures. The structure validation results for the two original and the two re-refined structural ensembles are shown in Table 1.

**Table 1:**
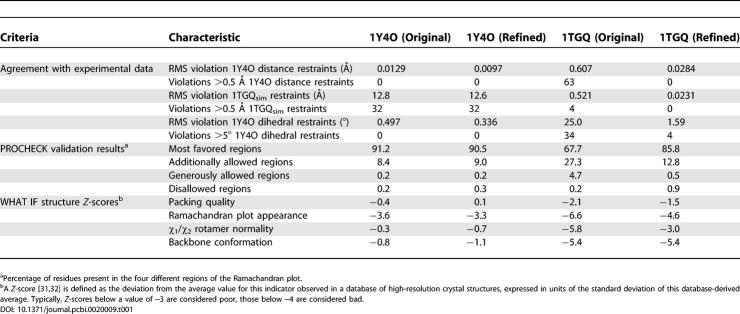
Average Quality Indicators of the 1Y4O and 1TGQ Structure Ensembles before and after Refinement in Explicit Solvent

The 1Y4O ensemble demonstrates a good agreement with the experimentally deposited restraints. For the distance restraints, no violations larger than 0.5 Å are observed, for the dihedral angle restraints, we find no violations larger than 5°. Both these thresholds are widely considered as compatible with and representative for a good structure within the NMR community. As expected, the 1TGQ_sim_ dataset of erroneous intramolecular restraints exhibits very large violations for the 1Y4O ensembles. The validation scores, as determined by the programs PROCHECK [29] and WHAT IF [30], all fall within acceptable ranges; only the Ramachandran plot *Z*-score [31] of −3.3 might be considered poor [32]. Still, this score is substantially better than that of a typical NMR structure taken from the PDB [8]. The refinement in explicit solvent slightly improves the quality indicators of the 1Y4O ensemble and the agreement of the structures with the experimental data. For comparison, we also evaluated of the quality of the recently released and the updated DLC2A entries in the PDB (entries 1Z09 and 2B95, respectively). Both exhibit quality scores much comparable to those of the 1Y4O ensemble, with again only the Ramachandran plot score being somewhat poor (data not shown).

The quality indicators for the deposited 1TGQ ensemble are, however, considerably worse when compared to those of the 1Y4O structure models: the majority of the quality *Z*-scores identify this structure as an outlier (*Z*-score < −4). The agreement of the original 1TGQ ensemble with the experimental restraints from 1Y4O is quite poor, but this is to be expected as these restraints were not used in the actual 1TGQ structure determination. The agreement of the 1TGQ_sim_ dataset with the 1TGQ ensemble is much better than for the 1Y4O ensemble. After a refinement in explicit solvent, the 1TGQ ensemble has accommodated to all distance restraints and does not show any violations larger than 0.5 Å. It is, however, unable to completely fulfill the experimental dihedral angle restraints of the 1Y4O dataset. On average four dihedral angle restraints per structure are violated by more than 5° in the refined 1TGQ ensemble, but none of these violate more than 15°. The refinement results in a considerable improvement of the PROCHECK validation scores and the percentage of residues in the most favored regions of the Ramachandran plot increases to a commonly considered acceptable score of 85.8%. Most of the WHAT IF quality *Z*-scores improve, but both the Ramachandran plot and the backbone normality scores remain at a very worrisome level (below −4). Also the χ_1_/χ_2_ rotamer normality does not reach the level of quality typically observed for this quality indicator after a refinement in explicit solvent [8].

All in all, our results show that an incorrectly folded NMR structure is easily refined to a good agreement with the experimental input data and acceptable PROCHECK Ramachandran plot statistics. The overall WHAT IF quality indicators identify the structure as problematic, but only the χ_1_/χ_2_ rotamer normality score is significantly worse than the 100 refined structures present in the DRESS database [8]. When judged by its overall quality parameters, it is understandable, but nevertheless worrisome, that the erroneous 1TGQ ensemble went unnoticed through the structure determination and validation pipeline at the NESGC. However, a more detailed inspection of the validation results shows that the problematic regions of this ensemble of structures could have been identified.

### Structure Validation on a per-Residue Basis

One of the first and very straightforward indicators that something might be wrong with the 1TGQ structure ensemble is the large discrepancy between the predicted and observed secondary structure, as shown in Figure 1A. Modern secondary structure prediction algorithms, such as the PSIPRED algorithm [33] applied here, typically yield predictions with an accuracy of 75% to 80%. The large deviations between predicted and observed secondary structure for the α2, β3, and β4 regions justify a further detailed inspection of these parts of the protein.

Figure 2 shows the per-residue scores of the two refined ensembles for four different WHAT IF quality indicators. The refined 1TGQ ensemble exhibits lower values for the packing quality [34] (see Figure 2A) compared to the refined 1Y4O ensemble, most notably in the α2, β4, and β5 regions. When the packing quality scores of 1TGQ are evaluated by themselves, the problematic regions do not particularly stand out. The same notion holds for the rotamer normality *Z*-scores (see Figure 2C), although the continuous stretch of residues from Pro45 to Arg80 with relatively low-quality scores should be considered suspicious. This is also expressed in the lower overall rotamer normality score, as already shown in Table 1. A nearly identical stretch of low scoring residues (from Met55 to Ile85) is observed when evaluating the Ramachandran plot quality scores (see Figure 2B). The finding that similar regions of consecutively low scoring residues are highlighted by different quality indicators provides more circumstantial evidence of the underlying problems, but again, no exceptional outliers are found.

**Figure 2:**
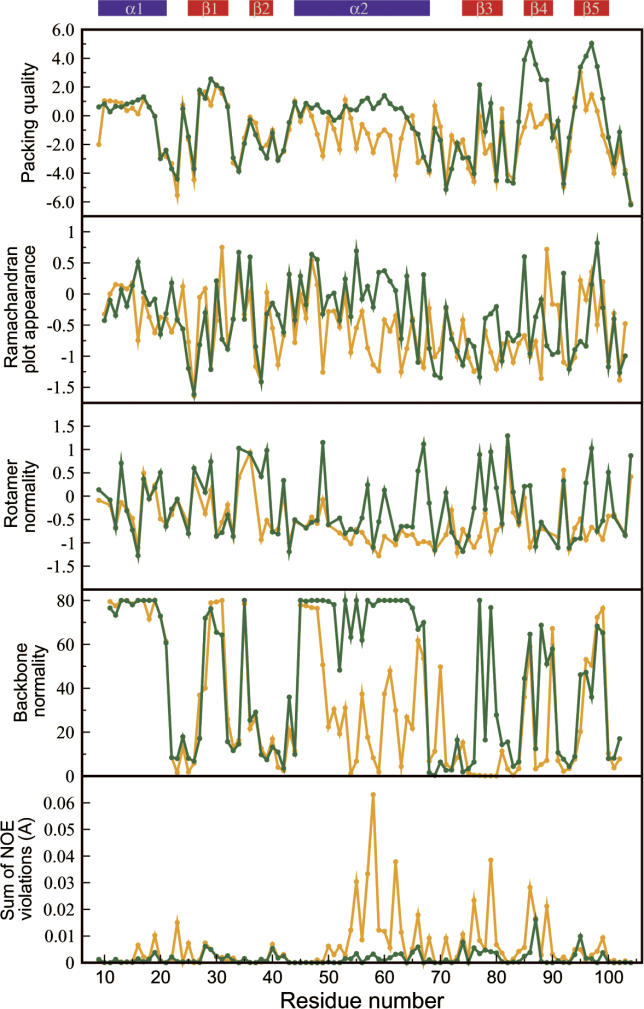
Five Different per-Residue Structural Quality Indicators (A) Packing quality *Z*-score. (B) Ramachandran plot appearance *Z*-score. (C) Rotamer normality *Z*-score. (D) Backbone normality score. The values listed on the *y*-axis indicate the number of times the local backbone (defined by the current residue plus or minus two residues) was found in WHAT IF's internal database (with a cut-off on the number of hits at 80). (E) Sum of the NOE violations. Scores for the refined 1Y4O ensemble are shown in green; those for the refined 1TGQ ensemble are shown in orange. Secondary structure of the 1Y4O ensemble is indicated using colored boxes: α-helices are shown in blue, β-strands are shown in red.

Our analysis shows that only the backbone normality score unambiguously identifies the erroneous regions in the 1TGQ structure ensemble. Figure 2D shows the number of occurrences of the local backbone conformation of each residue in WHAT IF's nonredundant internal database. For NMR structures, it is quite common to find low backbone normality scores in loops and other flexible regions, as evidenced by the validation results of the 1Y4O ensemble where most low scoring regions are found between the different secondary structure elements. These low scoring loops do, however, not influence the overall backbone normality score, which for the 1Y4O structures falls well within the normal range (Table 1).

Regular secondary structure elements, such as α-helices, typically score very well on the backbone normality check (e.g., the α1 region in both ensembles and the α2 region of 1Y4O). In the 1TGQ ensemble, however, unusually low backbone normality scores are observed for most residues in the α2 region. A near-zero number of hits is obtained for several residues (e.g., Met54, His55, Leu59, and Ser63), most of which are involved in bending the α2-helix. Alarming are the successive residues Thr75-Arg80, which all have a backbone occurrence score of 0, indicating that no similar backbone conformations are observed in the WHAT IF internal database of high-quality crystal structures [35]. This is not uncommon for occasional residues in loops but highly unlikely for consecutive residues in a well-defined region of the structure and is indicative of either a very unique or a very wrong backbone conformation. In either case, these results indisputably warrant an in-depth investigation of these regions of the structure and the experimental data that define them.

To assess if the experimental data also indicate the same regions as problematic, the sum of the NOE violations per residue is shown in Figure 2E. The found violations are small and would under normal circumstances not be considered problematic, but again they are clustered in the α2/β3 region. To further investigate this finding, we also analyzed the dataset constructed for the 1TGQ ensemble using the QUEEN program [36]. Using a representation of the structure in distance space and concepts derived from information theory, QUEEN can quantify the information contained in both individual restraints and sets of restraints. For the 1TGQ dataset, the total information content *(I_total_)* and, for each of the individual restraints, the unique information content *(I_uni_)* and the average information content *(I_ave_)* were determined. We previously showed that combining the unique and average information content can be very useful in the identification of problematic restraints in an experimental dataset [36]. The *[I_uni_*,*I_ave_]* plot shown in Figure 3 clearly illustrates the varying information content of the different restraints in the 1TGQ dataset. Similar to previous work [37], we evaluated the 30 most important and most informative restraints, all located above the dashed line in Figure 3. In total, 13 of the 30 most crucial restraints (indicated by the black squares in Figure 3) are located in regions of the structure ensemble that score low on the backbone normality check. As such, an analysis of the 1TGQ dataset using QUEEN would also have highlighted the α2 and β3 regions as parts of the molecule deserving further investigation.

**Figure 3:**
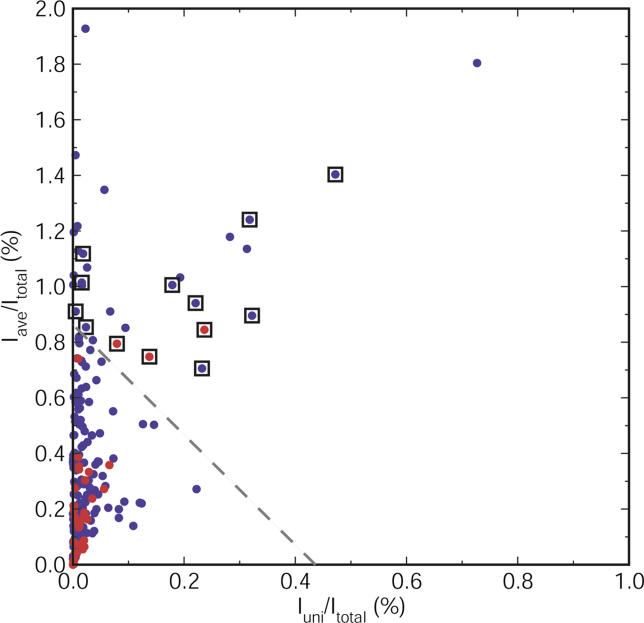
*[I_uni_,I_ave_]* Plot for 1TGQ Calculated Using the QUEEN Program [36] Long-range restraints (blue filled circles) and the 1TGQ_sim_ restraints (red filled circles) are indicated. Restraints that are among the 30 most unique and most important (those above the dashed gray line) and that involve residues in either the α2 or β3 region (cf. Figure 1A) are indicated by black boxes.

In summary, our analyses of both the structure ensemble and the supposedly observed experimental data of PDB entry 1TGQ clearly reveal the erroneous regions present in this set of structural models. Such a severe error therefore should not have gone undiscovered in any structure determination project.

### Evaluation of a Large Set of Recent NMR Structures

The fact that the erroneous 1TGQ ensemble made it into the PDB inevitably raises the question if more comparatively large errors might have gone unnoticed. To answer this question, we performed a quality analysis of a large set of protein NMR structures, the results of which are shown in Figure 4. The presented dataset was constructed by selecting from the PDB all NMR structures that were deposited after January 2003, consisted of at least 45 amino acids, and had more than 40% of their amino acids involved in secondary structure elements. The latter criterion was imposed to remove the models of largely unfolded structures that might bias our analysis. From this set all structural genomics target were filtered (310 in total), their quality scores are shown in orange in Figure 4. From the remaining NMR structures, originating from individual structure determination laboratories, an equally sized random selection of structures was made, whose quality scores are shown in green in Figure 4. For comparison, the average quality scores of the 1TGQ ensemble, both before and after refinement, are also indicated.

**Figure 4:**
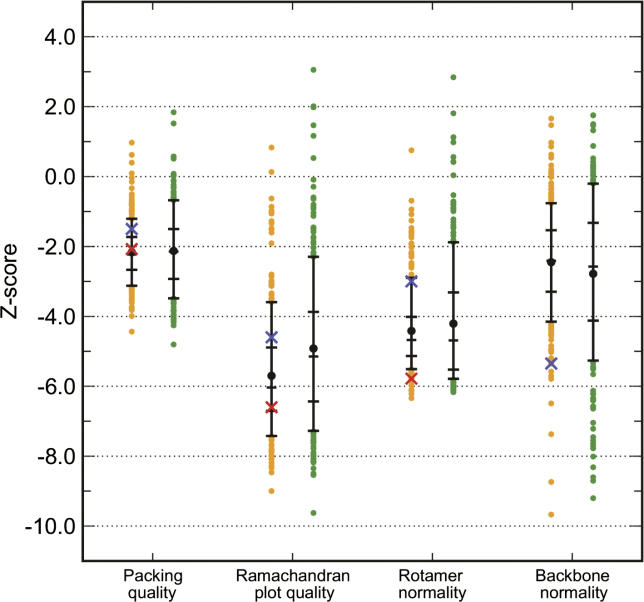
Structure Quality *Z*-Scores for a Large Set of Recent NMR Structures The quality scores of 620 NMR ensembles released from the PDB after January 1, 2003, are shown. For comparison, the dataset is separated in structures solved as part of structural genomics projects (orange) and structures originating from individual research groups (green). For each quality indicator, the average *Z*-score is indicated with a filled black circle. The black horizontal markers indicate (from top to bottom) the 90th, 75th, 50th (the median), 25th, and 10th percentiles of the data points for each quality indicator. The distribution of the outliers outside the markers is indicated using colored data points. The quality scores of the original and refined 1TGQ ensemble (cf. Table 1) are indicated by red and blue crosses, respectively. The backbone normality score of 1TGQ is identical for the original and refined ensemble.

The data show no significant difference between the distributions of the quality indicators of structural genomics structures compared to those structures originating from individual research groups. In general, the distribution of the quality scores appears to be somewhat narrower for the structural genomics structures, but the average scores are similar, a result in-line with recent other studies [38]. Surprisingly, for both the packing and Ramachandran plot quality scores, the 1TGQ ensembles score comparable to the majority of the NMR structures. The rotamer normality score initially places the 1TGQ ensemble among the 10% worst scoring structural genomics structures, but after refinement it is amidst the top 10%. As before, the backbone normality score consistently identifies the erroneous 1TGQ structures as one of the outliers. Given the serious errors present in the 1TGQ ensemble, one might consider the fact that several NMR structures solved over the past years demonstrate backbone normality scores lower than those of 1TGQ rather worrisome.

Visual inspection of the structural ensembles exhibiting lower backbone normality scores than 1TGQ revealed that in some instances these low scores resulted from the corresponding proteins exhibiting unusual folds or dynamic behavior. For others, however, we noted some striking structural abnormalities of which we will discuss two examples. First, our attention was drawn to the NMR structure with the lowest backbone normality *Z*-score (*Z* = −9.8). It corresponds to an alternatively spliced PDZ domain of PTP-Bas [39] (PDZ-Bas, PDB entry 1Q7X), which was determined in the context of the Structural Proteomics In Europe project (SPINE, http://www.spineurope.org). In this structure ensemble, an arginine side chain deeply penetrates the hydrophobic core (cf. Figure 5A). Arginine, however, is a very hydrophilic residue and is typically not observed in hydrophobic environments. In the highly identical alternative spliced second PDZ domain of PTP-BL [37] (PDZ-BL, PDB entry 1OZI, sequence identity 95% with PDB entry 1Q7X) and, to the best of our knowledge, in all other homologous PDZ domains, the corresponding arginine is indeed solvent exposed (cf. Figure 5B), rendering it very unlikely for the 1Q7X ensemble to be correct. This finding is corroborated by the backbone residual dipolar coupling (RDC) data [40] measured for the PDZ-BL protein [37]. To allow for a fair comparison, an ensemble of 20 PDZ-BL structures was calculated and refined using only the experimental distance and dihedral data and the procedures described above, as the deposited structures [37] were refined against the RDC restraints. The RDC R-factor [41] obtained for the newly calculated PDZ-BL ensemble is 43%, whereas the RDC R-factor of 69% for the PDZ-BAS ensemble is significantly higher. This clearly demonstrates the ability of RDC-derived orientational restraints to also distinguish incorrect backbone orientations, but unfortunately these data are typically not acquired in structural genomics pipelines.

**Figure 5:**
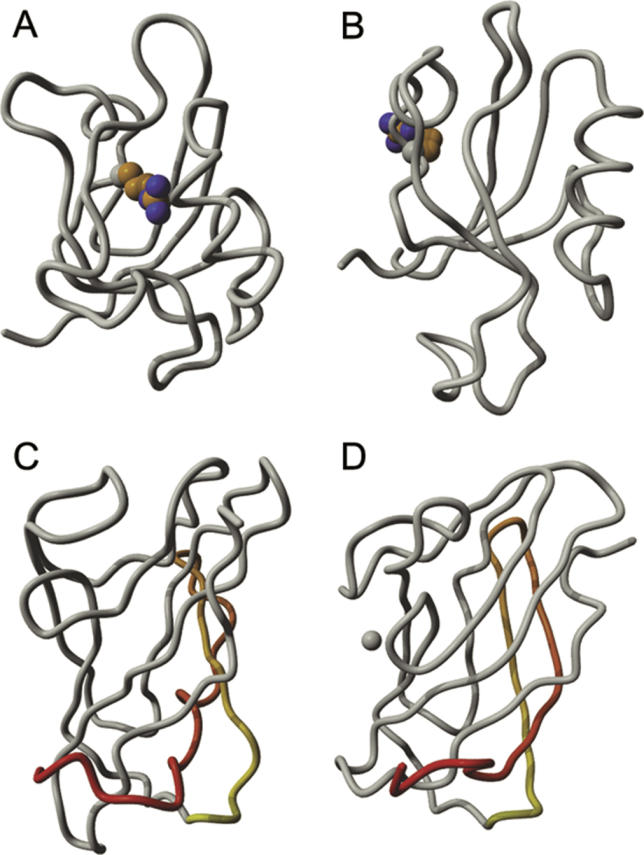
Examples of Observed Structural Anomalies (A) An arginine side chain protruding the hydrophobic core of the second PDZ domain of PTP-Bas [39]. (B) The corresponding arginine in the highly homologous second PDZ domain of PTP-BL [37] is solvent exposed. (C) The C-terminal region of DR1885 [42] (residues 120 to 149 are color-coded from yellow to red) forms a knot-like structure in the apo-form of DR1885. (D) In the copper bound form of DR1885, the C-terminus wraps around the protein, instead of traversing through it. For each of the four structure ensembles, only the first, and presumably best, model is shown.

As a second example, we noticed striking differences between the apo- and copper bound forms of the protein DR1885 [42] (PDB entries 1X7L and 1X9L), also originating from the SPINE project. Most notable are the differences in the conformation of the C-terminal region of the protein (residues 120 to 149, Figure 5C and 5D). In the apo-form these residues are in a very unusual knot-like conformation, with the C-terminus passing through a loop consisting of residues 118 to 125. In the copper bound structures, the backbone of the C-terminal residues assumes a much more normal conformation and wraps around the DR1885 protein, instead of traversing through it. Given that there are no significant changes in the chemical shifts of the residues involved upon binding of copper to DR1885 (see Figure 2C in [42]), one of the two structure ensembles is almost certain to be incorrect.

In the publications describing the DR1885 protein [42] and the alternatively spliced PDZ domain from PTP-Bas [39], structural quality is foremost assessed by the number and size of the restraint violations and PROCHECK Ramachandran plot statistics. Our findings for the DLC2A protein already illustrated that these quality indicators are relatively insensitive to large structural errors, a result corroborated by the relatively acceptable scores found for these two datasets. Therefore, these examples clearly illustrate that the fact that no distance or dihedral angle violations are observed above a given threshold and that majority of the residues are found in allowed regions of the Ramachandran can be indicative of a good structure but does not provide any guarantees. It is interesting to note here that the three erroneous structures described in this paper stem from premier protein NMR groups, all involved in the development of structure validation and refinement methodologies [43–46], and that these methodologies either failed or were not or incorrectly applied in identifying the serious errors present in these structure ensembles.

To hopefully prevent such large errors from reoccurring in the future, we strongly suggest that validation results from normality checks, such as those implemented in the WHAT IF program [4,30], should be evaluated (and reported on) in any structure determination project. For high-throughput structural genomics projects, the application of multiple and sophisticated validation tools is even more critical, as much effort is geared towards minimizing the amount of expert time required for the determination and refinement of NMR structures [47]. Since this amount is deliberately continuously reduced, we expect structural genomics projects to become increasingly dependent on data and structure validation software to direct the spectroscopist to the regions that warrant his or her expert assessment.

### Conclusions

We have shown that, when using only distance and dihedral restraints, even a largely incorrect structure is readily refined to seemingly acceptable levels of quality. As a result, the quality of biomolecular NMR structures cannot be safely assessed by the size and number of residual restraints violations, the precision of the structure ensemble, or even the fact that most residues are located in the allowed regions of the Ramachandran plot. Relying solely on these indicators to evaluate an ensemble of NMR structures therefore provides a false sense of correctness. The fundamentally different nature of residual dipolar couplings renders them complementary to traditional NMR data and a powerful tool to identify large errors in NMR structures. Unfortunately, in many instances, such as in most structural genomics efforts, they are not routinely acquired and proper use of structure validation tools then becomes crucial. Furthermore, our results show that also more sophisticated quality indicators, e.g., the overall WHAT IF backbone normality score, do not unambiguously identify problematic structures. In contrast, we showed that only the simultaneous evaluation of multiple quality indicators on a per-residue basis, however, combined with a careful evaluation of the experimental data (e.g., using QUEEN), does allow for the well-supported identification erroneous regions in biomolecular NMR structures, thereby avoiding errors as those reported here.

## Materials and Methods

### NMR structures and data.

For both mDLC2A and hDLC2A, the structure ensembles were obtained from the PDB (PDB entries 1Y4O and 1TGQ, respectively). The residue numbering of the 1TGQ ensemble was adjusted to match to that of the 1Y4O ensemble, as shown in Figure 1. The coordinates describing the His-tag in the 1Y4O ensemble (residues 1 to 8) were removed so that all DLC2A models contained an equal number of residues.

The experimental restraints for the 1Y4O ensemble, solved as a dimer, were obtained from the PDB, for the 1TGQ ensemble no experimental restraints were available at the time of writing. All stereospecifically assigned NOEs were deassigned for the violation analyses, structure calculations, and refinements. To be able to apply the same dataset to both structures, all restraints involving unique atoms of the three amino acids that are different in both sequences (cf. Figure 1A and 1B) were removed from the dataset. The final dataset contained 1,395 distance restraints, consisting of 553 intraresidue, 341 sequential, 278 medium-range, 187 long-range, and 72 intermolecular restraints. In addition, 146 dihedral angle restraints were included in all refinements. The deposited dataset also contained 96 hydrogen bond restraints, but as it is not clear how these were derived, and as they showed considerable violations in the deposited 1Y4O ensemble, these restraints were excluded from all analyses.

### Structure calculation and refinement protocols.

All structure calculations were performed using CNS [48] and the default simulated annealing protocol, as provided with the software package. All refinements in explicit solvent [7] were performed using XPLOR-NIH [49] using the refinement procedure as described before [8]. Both the deposited and newly generated structure ensembles were validated using PROCHECK [29] and WHAT IF [30]. The deposited and constructed datasets were evaluated using the QUEEN program [36].

## Supporting Information

### Accession Numbers

The UniProt (Universal Protein Resource) (http://www.pir.uniprot.org) accession numbers for mDLC2A and hDLC2A are P62627 and Q9NP97, respectively.

## Abbreviations

DLC2A: dynein light chain 2A
NMR: nuclear magnetic resonance
NOE: nuclear Overhauser enhancement
PDB: Protein Data Bank
RDC: residual dipolar coupling
